# Moonshine.jl: a Julia package for genome-scale model-based ancestral recombination graph inference

**DOI:** 10.3389/fgene.2026.1753780

**Published:** 2026-04-13

**Authors:** Patrick Fournier, Fabrice Larribe

**Affiliations:** Département de Mathématiques, Université du Québec à Montréal, Montréal, QC, Canada

**Keywords:** algorithms, ancestral recombination graph, ARG inference, coalescent theory, Julia

## Abstract

The ancestral recombination graph (ARG) is the model of choice in statistical genetics to model population ancestries. Software capable of inferring ARGs on a genome scale within a reasonable amount of time are now widely available for most practical use cases. While the inverse problem of inferring ancestries from a sample of haplotypes has seen major progress in the last decade, it does not enjoy the same level of advancement as its counterpart. Up until recently, even moderately sized samples could only be handled using heuristics. In recent years, the possibility of model-based inference for datasets closer to ”real world” scenarios has become a reality, largely due to the development of threading-based algorithms. This article introduces Moonshine.jl, a Julia package that has the ability, among other things, to infer ARGs for samples of thousands of human haplotypes of sizes on the order of hundreds of megabases within a reasonable amount of time. On recent hardware, our package is able to infer an ARG for samples of densely haplotyped (over one marker/kilobase) human chromosomes of sizes up to 10,000 in well under a day on data simulated by msprime. Scaling up simulation on a compute cluster is straightforward since each ARG is inferred independently using a single thread. While model-based, it does not resort to threading but rather places restrictions on probability distributions typically used in simulation software in order to enforce sample consistency. In addition to being efficient, a strong emphasis is placed on ease of use and integration into the biostatistical software ecosystem.

## 1 Introduction

Coalescent theory is the framework of choice when it comes to inference from genetic data (Nordborg, 2007). A brief introduction and some historical notes on the subject can be found in Lewanski et al. (2024). Although it originates from the seminal papers of Kingman (1982a), Kingman (1982b) and Hudson (Hudson, 1983), it did not really gain widespread use before the advent of computer software capable of simulating the coalescent process with recombination. ms (Hudson, 1983), a software written in C, was the first of its kind. It aims at generating a sample of genetic sequences evolving under a Wright-Fisher (WF) model *via* a coalescent-with-recombination (CWR) approximation. The WF model is known for its simplicity. Somewhat surprisingly, simulating a sample of genetic sequences resulting from the evolution of a population obeying it rapidly becomes a challenging task. This is because it is necessary to keep track of every sequence at each of the numerous discrete steps of the simulation procedure from the original population to the desired sample. The coalescent process works in reverse: it starts from the sample and generates coalescence events, which correspond to sets of sequences finding a common ancestor. Consequently, it is only necessary to keep track of haplotypes associated with these events. The remaining sequences are deemed *non-ancestral* for the sample and can be disregarded. In addition, exponentially distributed coalescence times are substituted to discrete generation counts. Sampling genetic sequences using the coalescent approximation is a straightforward task.

What is not trivial, however, is relaxing the assumptions of the WF model to allow recombination events. The result of simulations performed under a model accounting for these events, along with, somewhat confusingly, the statistical model itself, is known as the ancestral recombination graph (ARG). To avoid ambiguity, we reserve “ARG” for the former and refer to the statistical model through “CWR”. While Hudson’s ms is able to simulate recombination events, it is not efficient enough to meet the requirements of large-scale genomic data analysis. To reduce the computational burden associated with simulations, an approximation to the CWR was developed by McVean and Cardin (2005). Building on the work of Wiuf and Hein (1999), their idea is to approximate the non-Markovian recombination process with a Markovian one called the sequential Markov coalescent (SMC). This gave rise to a plethora of so-called sequential simulators such as MaCS (Chen et al., 2009), fastsimcoal (Excoffier and Foll, 2011), SC (Wang et al., 2014) and scrm (Staab et al., 2015). While the sequential approach is not inherently more efficient than the backward-in-time one, it is easier to approximate, leading to substantial improvements in the capacity of CWR software. Nonetheless, the idea of backward-in-time simulation was not abandoned, as evidenced by MSMS (Ewing and Hermisson, 2010) and Discoal (Kern and Schrider, 2016). Messer (2013) even developed a forward-in-time simulator able to handle non-WF scenarios. Some years later, the classical backward-in-time CWR approach was brought back into the spotlight by msprime (Kelleher et al., 2016). By reformulating Hudson’s original ms in terms of a data structure they called *sparse trees*, their Python package (with performance-critical procedures implemented in C) has the capacity of simulating exactly from the coalescent with recombination more efficiently than the sequential approximations available at the time. Version 1.0 (Baumdicker et al., 2021) brings even more features such as the ability to simulate directly from a WF model. For all these reasons, msprime is nowadays the *de facto* reference for CWR and even ARG simulation.

Programs mentioned above simulate genealogies with the goal of generating a sample of genetic sequences. Throughout this paper, we refer to this process as *ARG simulation*; the ancestry is viewed as a sample point in the probability space associated with the CWR. The focus is generally on inferring parameters such as recombination/mutation rates or effective population size. The distinctive characteristic of this class of algorithms is that the distribution is parametrized by values derived from sequences of markers rather than the markers themselves. This is to be contrasted with software designed to solve the inverse problem of generating *likely* ancestries, or even, in some cases, a single ancestry directly from the markers. This is known as *ARG inference* since the ancestry itself is usually the main point of interest. This choice of words is, however, somewhat misleading as so-called inferred genealogies are not necessarily central to the analyses they are involved in. They can be instrumental in the estimation of other parameters, just like their simulated counterparts. It is true that their distribution is not that of the CWR. That being said, this is not to say that alternative likelihood functions enabling maximum likelihood estimation do not exist. Indeed, recent work (Bisschop et al., 2025) proposes formulations with applicability to broad classes of ancestries in mind. While the problem these pieces of software solve is by nature more computationally demanding, they provide major benefits in that the ancestries they produce are generally more likely with respect to the sample at hand. In particular, their usefulness cannot be overstated for methodologies that aim at improving the estimation of parameters by treating the ancestry of a sample as a latent variable such as Larribe et al. (2002). Those require integrating these genealogies out, a task involving, in practice, the ability to sample in high probability regions.

Early attempts at ARG inference include recom (Griffiths and Marjoram, 1996) and Infs (Fearnhead and Donnelly, 2001). More recently, the authors of SC also provides a modified version of their algorithm called *SC-sample*, capable of solving this inverse problem by generating sample-consistent graphs. A graph is said to be consistent for a sample with respect to a mutation evolution model if it generates the sample under that model. In Wang et al. (2014), they choose to use the popular infinite site model (ISM). Other software have been developed with the goal of inferring the ancestry of a sample. ARGinfer (Mahmoudi et al., 2022) assumes a sample of sequences of binary markers evolving under the ISM. It aims at performing the inference probabilistically *via* a MCMC scheme allowing, for instance, computation of probability intervals for ARG-dependant quantities. Another noteworthy inference software is ARGweaver Rasmussen et al. (2014), which implements an ancestry reconstruction algorithm based on the progressive integration of sequences to an existing ARG, an operation the authors call *threading*. This approach forms the basis for recent methods such as SINGER Deng et al. (2025). We should also mention ARBORES Heine et al. (2018), a new take on the *tree scan* methods Song and Hein (2005) and Espalier, which implement its own original algorithm. These methods are designed to reconstruct ARGs according to a probabilistic model. For that reason, we refer to them as being *model-based*.

A related problem is that of *parsimony-based inference*, which consists of finding ARGs consistent with a sample using the minimum number of recombination events. This problem has been proved NP-hard (Wang et al., 2001). Nonetheless, attempts to solve it exactly and approximately date back to the early work of Hein (1990), which was improved by Song and Hein (2005). One of the first widely available programs for parsimony-based inference is Margarita Minichiello and Durbin (2006), which later inspired ARG4WG Nguyen et al. (2017) and GAMARG Thao and Vinh (2019). SHRUB Song et al. (2005) and beagle Lyngsø et al. (2005) implement algorithms sharing similarities with that of Margarita although they were developed independently. The more recent KwARG has many features reminiscent of these two programs. Similar to ARBORES, RENT Wu (2011) and its successor RENT+ Mirzaei and Wu (2016) are based on the tree scan method. TMARG implements two algorithms: one is related to SHRUB’s while the other derives from the notion of *Steiner sequence*. SARGE Schaefer et al. (2021) implements a greedy algorithm built on top of the four gametes test Hudson and Kaplan (1985).

Some methods are partially founded on statistical frameworks, but employ heuristics at various levels. This is the case for tsinfer Kelleher et al. (2019), Relate Speidel et al. (2019), and Threads Gunnarsson et al. (2024), which are based on the Li-Stephens model Li and Stephens (2003). Specifically, tsinfer uses a bespoke consensus heuristic to infer ancestral haplotypes, Relate estimates the topology of marginal trees *via* hierarchical clustering and Threads uses a threading algorithm that involves matching haplotypes through a method based on the positional Burrows–Wheeler transform Durbin (2014). ARG-needle Zhang et al. (2023) relies on the Ascertained Sequentially Markovian Coalescent Palamara et al. (2018) for pairwise coalescence times estimation of subsets of sample haplotypes constructed by genotype hashing.

This paper introduces a Julia package called Moonshine implementing inference of sample-consistent ancestral recombination graphs. The approach is sequential, as it is based on iterative modification of the ARG. A sequence of operations are applied to a coalescent tree to ultimately make it consistent with a sample of haplotypes. The main advantage over back-in-time approaches is the possibility it offers users to specify various levels of approximation when generating ARGs. The available spectrum ranges from *exact* simulation without any Markov assumption to first-order approximation *à la* SMC. In any case, the object resulting from the ARG construction routine is the same, regardless of the level of approximation. It contains the whole graph, as well as meta-data such as vertex latitudes (number of generations from the sample), associated haplotypes, and intervals of ancestrality for edges, all available to users for subsequent analysis. In fact, Moonshine is designed for easy integration into data analysis workflows. In addition, it is possible to use it for inferring a set of ancestries consistent with a given sample of genetic sequences. It can be used interactively without sacrificing performance, thanks to Julia’s just-in-time compilation. It is also fully integrated with Julia’s ecosystem of graph theoretical packages. Although all sequential procedures are based on the same idea and, consequently, share many similarities, Moonshine implements its own original algorithm. Furthermore, since it is created with statistical inference in mind, Moonshine treats ARGs as random graphs. It is straightforward to evaluate ARG-related functions such as probability densities for the ARGs themselves or other random variables, such as phenotypes, conditional on an ARG. Implementation of custom functionalities is facilitated by a coherent type hierarchy and thorough documentation of abstract types and interfaces, making the extension of the package’s various components as easy as possible. Finally, interoperability with tskit (Kelleher et al., 2016; Wong et al., 2024) streamlines data management. Generating random samples directly from tskit objects is supported, and a convenience constructor for obtaining a sample from a simple genetic model using msprime (Baumdicker et al., 2021) is provided. This is transparent to the end user, thanks to Moonshine being packaged with its own distribution of msprime. Additionally, the ARGs produced by Moonshine can be converted into TreeSequences with a single function call. Conversely, Moonshine can be installed and used entirely from Python *via* the packages JuliaPkg and JuliaCall, respectively.

Moonshine shares with SC-sample the capacity of performing inference under various levels of approximation. To the best of our knowledge, these are the only two model-based inference methods implementing this functionality; ARBORES, ARGWeaver, Singer, and Espalier are based on SMC while ARGinfer does not allow for approximation. Allowing for approximate inference increases scalability by enabling a trade-off between biological realism and computational performance. On the other hand, Moonshine shares with SC-Sample and ARGinfer the ability to produce ancestries with complex correlation structures that may include type 2 recombination events (see Subsection 2.2.1). Moreover, Moonshine supports multiple crossing over events (MCO), a unique feature among inference software. Unlike ARBORES, ARGweaver, SINGER and ARGinfer, Moonshine does not rely on MCMC and is thus fast and easy to use. No iterations are lost in burn-in periods or thinning intervals, and there is no need for convergence diagnostics or parameter tuning.

Our objective in developing Moonshine is not limited to creating a realistic and convenient ARG inference software; performance is a major priority. We present numerical experiments showing its potential for both coalescent tree construction and ARG inference. Trees for large samples (n=10000) of long simulated haplotypes (250 Mbp) can be constructed in minutes at high resolution (over one marker per kbp) using Hamming distance between sequences. In the same scenarios, complete ancestries can be inferred in hours. Furthermore, our algorithms are completely single-threaded, enabling us to increase sampling throughput by leveraging concurrency efficiently and easily by launching multiple ARG inferrence instances in parallel. By being faster than other model-based alternatives, Moonshine represents a step towards enabling probabilistic inference of ancestries on the genome scale.

## 2 Methods

### 2.1 Tree construction

Similar to other sequential procedures, the first step of our algorithm is to construct an initial coalescent tree. Consistency with the first marker is not assumed; ARG inference can be carried out even starting from a completely random tree. The sole requirement is that it be a valid coalescent tree, i.e., a full binary tree with a coherent set of latitudes for the vertices. The idea behind this functionality is that it might be of interest to compare the performance of a method under a model without recombination *versus* one that allows for such events. It would make little sense in that context to give a special status to a single marker, disregarding the remainder of the haplotypes. Consequently, we give the user maximum flexibility when building coalescent trees, which may be of interest both in their own right and as a stepping stone for constructing more complex histories. As will be discussed later, Moonshine is packaged with two haplotype metrics designed for tree building. It is straightforward for the user to implement custom metrics.

Within our package, data structures representing genealogies are subtypes of the AbstractGenealogy abstract type. The type of coalescent trees is simply Tree. Given a sample (of type Sample) of phased and polarized genetic sequences (of type Sequence), construction is controlled by two parameters: the global mutation rate μ and a metric on haplotypes d . Assuming a diploid population, sequences of length l with s markers, a diploid effective population size $N_{e}$ and a constant per locus diploid mutation rate of $\mu^{\prime }$ , the global mutation rate is $\mu =4N_{e}\mu^{\prime }l$ . These parameters are either computed or explicitly passed to Sample’s constructor. As for the metric, since Moonshine is compatible with binary markers exclusively, a sample of size n is an n -tuple $H=\left(h_{1},\ldots ,h_{n}\right)$ of bit vectors of size s . Concretely, for each sequence k , we have

$$h_{k}=h_{k}^{1}\cdots h_{k}^{s}$$

where $h_{k}^{•}\in \left\{0,1\right\}$ . Wild and derived alleles are represented by 0 and 1 respectively as is standard in the literature. Let ⊕ denote addition modulo 2. Examples of useful metrics, also known as *distance functions*, include

$$\begin{array}{ll} d_{L}\left(h_{1},h_{2}\right) & =h_{1}^{1}⊕h_{2}^{1}\\ d_{H}\left(h_{1},h_{2}\right) & =\overset{s}{\underset{i=1}{\sum }}h_{1}^{i}⊕h_{2}^{i}. \end{array}$$

$d_{L}$ is the metric under which the distance between two sequences is zero if and only if the state of their first marker is identical, 1 otherwise. $d_{H}$ is the Hamming distance. Such discrete distances have a natural biological interpretation as the number of mutations between (a subinterval of) sequences. Arbitrary distances can be implemented by the user as subtypes of Distance.

Detail-oriented readers might have noticed that the term “metric” is used loosely, as the positivity axiom need not hold; the distance between two distinct haplotypes may be 0. This is necessary to allow inference of trees consistent for a single marker. Technically, the correct mathematical construct is that of a pseudometric.

#### 2.1.1 Construction algorithm

Coalescence events are generated as follows: a vertex $v_{a}$ is choosen uniformly among the set of *live* vertices, that is, the vertices that have not coalesced yet. Another vertex $v_{b}$ is selected conditional on $v_{a}$ . The probability $p_{ab}$ of selecting $v_{b}$ given $v_{a}$ is proportional to

$$p_{ab}=\frac{\mu^{d_{ab}}}{\Gamma \left(d_{ab}+1\right)}$$

where Γ is the gamma function, $d_{ab}=d\left(h_{a},h_{b}\right)$ is the distance function with $h_{a}$ and $h_{b}$ the haplotypes associated with $v_{a}$ and $v_{b}$ respectively. $p_{ab}$ is an unnormalized Poisson probability, where Γ is used instead of the usual factorial function to allow non-integer distances. Both vertices then coalesce into $v_{c}$ with associated haplotype $h_{c}=h_{a}⊙h_{b}$ where ⊙ is the Hadamard product. The shift in latitude is exponentially distributed with rate parameter equal to the number of live vertices. The latitude of $v_{c}$ is computed with respect to that of the previous event. This procedure replaces $v_{a}$ and $v_{b}$ by $v_{c}$ in the set of live vertices. Repeating it n−1 times on a sample of n haplotypes yields a coalescent tree.

The user has the possibility of biasing distance computation by specifying a parameter $c_{0}\in R_{+}\cup \left\{0,\infty \right\}$ meaning that, in practice,

$$d_{ab}=c_{0}d\left(h_{a},h_{b}\right).$$

$c_{0}$ can be used to reproduce the familiar behavior of other software. Setting $c_{0}=\infty$ and $d=d_{L}$ , we obtain

$$d_{ab}=\left\{\begin{array}{cc} 0 & \text{if }h_{a}^{0}=h_{b}^{0}\\ \infty & \text{if }h_{a}^{0}\neq h_{b}^{0} \end{array}\right.$$

resulting in

$$p_{ab}=\left\{\begin{array}{cc} 1 & \text{if }h_{a}^{0}=h_{b}^{0}\\ 0 & \text{if }h_{a}^{0}\neq h_{b}^{0} \end{array}\right..$$

As a result, haplotypes with identical status at the first marker will be agregated, resulting in a tree consistent with that marker.

**FIGURE 1:**
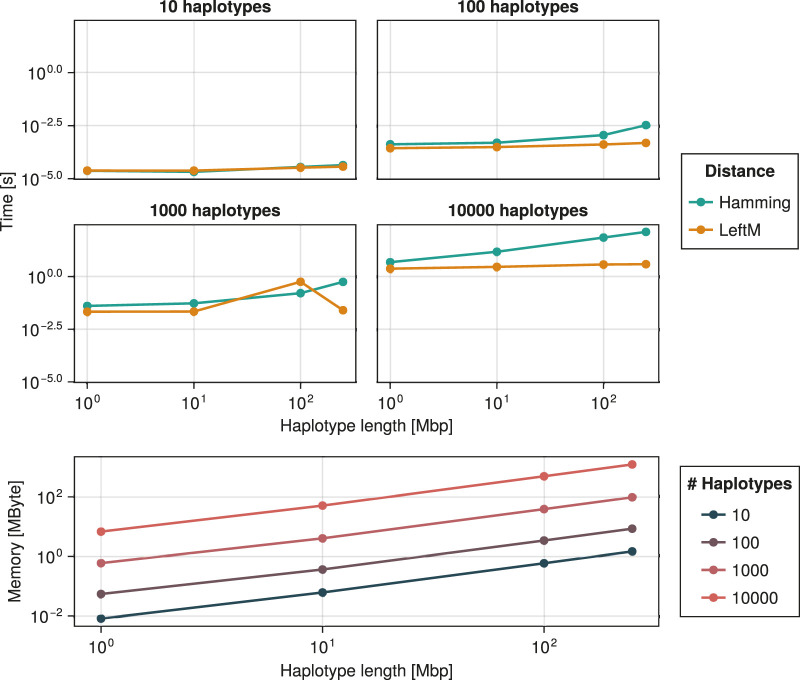
Time and memory needed for the construction of a coalescent tree as a function of the number of haplotypes, haplotype length, and distance function. Sampling is exact $\left(t_{0}=1\right)$ and no bias is applied ( $c_{0}=1$ ). Construction with respect to either of the two distances yields identical memory usage since their computation does not involve memory allocation.

**FIGURE 2:**
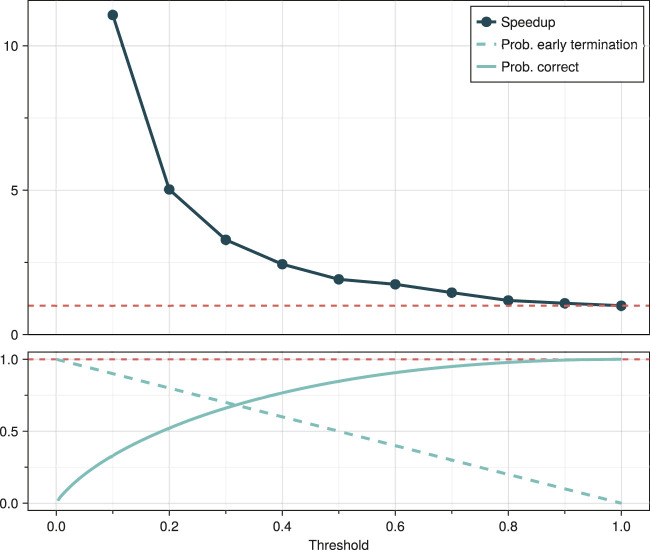
Speedup in tree construction, probability of selecting the correct sequence and probability of early termination as a function of the sampling threshold $t_{0}$ . No bias is applied $\left(c_{0}=1\right)$ . The probability of early termination is $1-t_{0}$ . As the number of candidate vertices increases, the probability of sampling correctly from the target distribution converges to $t_{0}\left(1-\log t_{0}\right)$ (depicted here). The exact probability for a single run is given by lemma 1.

In practice, dealing with a ratio of such extreme quantities poses a numerical challenge. For instance, if one wishes to use Hamming’s distance, computation of the normalizing constant becomes impossible even for relatively small values of n and s . We address these issues using two tricks. First, we compute $p_{ab}$ *via* Stirling’s approximation. On the logarithmic scale, we obtain

$$\log p_{ab}\approx d_{ab}\left(\log\mu -\log d_{ab}+1\right)$$

which is already more manageable. Next, it would be ideal to refrain from reverting to the linear scale. Moreover, we would greatly benefit from avoiding the computation of the normalizing constant altogether. It turns out that this is exactly what the so-called *Gumbel trick* Gumbel (1954) is designed to do. The trick transforms sampling from the target distribution into an optimization problem. For a set of candidate vertices $b_{1},\ldots ,b_{m}$ and a sequence of iid standard Gumbel random variables $g_{1},\ldots ,g_{m}$ , ${\text{arg max}}_{k}\left\{\log p_{ab_{k}}+g_{k}\right\}$ is distributed as a categorical random variable with the probability of category k being equal to $p_{ab_{k}}$ . This result gives us a more stable way of selecting the second coalescing vertex $v_{b}$ , at the cost of increased computing time for drawing Gumbel random variables and finding the maximum of the sequence. In many cases, this impact should be minimal compared to the overall execution time, for instance when the tree is to be used for ARG inference. Figure 1 provides a rough estimate of time and memory usage for various scenarios. For cases where time considerations warrant a precision tradeoff, users can sample approximately from the target distribution *via* a scheme we call *secretary sampling*, inspired by the famed *secretary problem* (Ferguson, 1989). It revolves around giving the algorithm a chance of terminating before having traversed the complete set of candidate vertices. Its behavior is controlled by a user-determined threshold parameter $t_{0}\in \left[0,1\right]$ . As illustrated in Figure 2, the probability of an early termination decreases with $t_{0}$ . Note that although normalization is not required, the unnormalized probabilities associated with traversed vertices must be summed in order to evaluate the density of the resulting coalescent tree. This has to be done carefully as departure from the logarithmic scale is unavoidable. Details as well as the complete algorithm are presented in Algorithm 1.

Algorithm 1 **Tree Construction.**

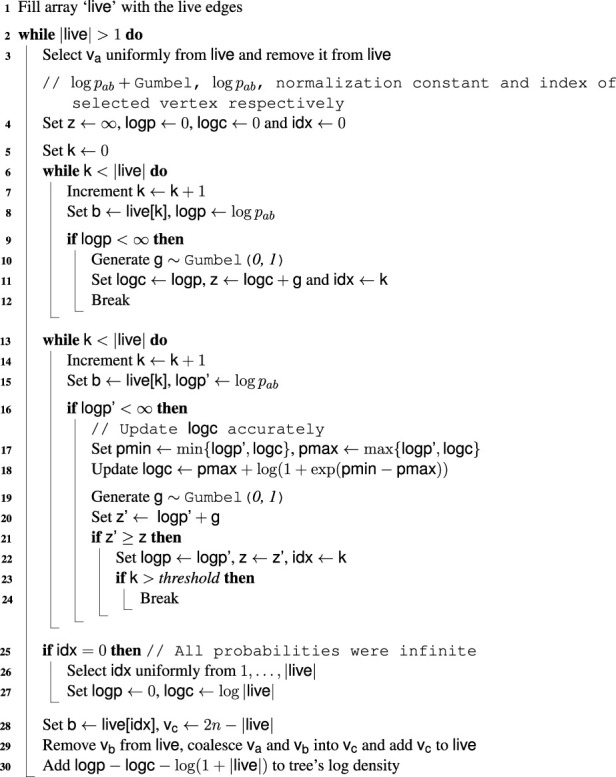

### 2.2 ARG inference

In Moonshine, ancestral recombination graphs are instances of the ARG type, a subtype of AbstractGenealogy. Since they represent a more realistic model for the ancestry of a sample subject to recombination, ARGs can be viewed as improved versions of coalescent trees. ARGs are constructed sequentially from a Tree by iteratively generating recombination events until an ancestry consistent with the sample is reached. As is common, consistency is defined through the ISM: a genealogy is consistent with a sample if the number of mutations per marker is at most one. In a context where the recombination rate is several orders of magnitude higher than the mutation rate, a consistent ARG is typically a more realistic genealogy than any other kind of inconsistent ancestry. Moonshine is consequently very well suited to working with single nucleotide polymorphisms (SNPs), which have a low mutation rate.

Ancestries are modified by recombination events, which partitions ancestral material into two subintervals: that to the left of an associated point, called a *breakpoint*, and the material to the right. From a graph-theoretical perspective, a recombination event is generally represented by a vertex of degree 3 with a single child and two parents. As an example, imagine that the child edge of a recombination vertex is ancestral for an interval I and that the associated breakpoint is b . In that case, one of the parental edges, generally referred to as the left edge, is ancestral for $\left.\left[0,b\right.\right)\cap I$ while the other (right) edge is ancestral for $\left.\left[b,\infty \right.\right)\cap I$ . Although we used the term “interval”, I can actually be a union of intervals. We will continue to use this terminology when the distinction between the two concepts is not relevant.

**FIGURE 3:**
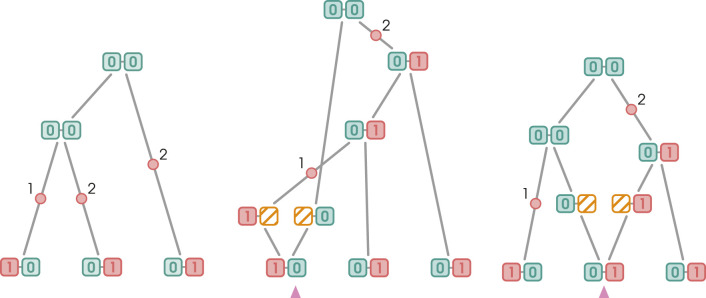
Two types of recombination events. Blue and red boxes represent SNPs with the wild and derived alleles, respectively. Non-ancestral material is represented by yellow boxes. Mutation edges are marked with red dots and numbers indicating the mutating markers. Pink arrows indicate the breakpoints. When recoalescence occurs *below* the MRCA of the sample (gMRCA), the recoalescence vertex is “inserted” by deleting an edge from the graph and replacing it with two new edges incident to the recoalescence vertex; this is illustrated in Figure 3. By contrast, no deletion is required when recoalescence occurs *above* the gMRCA; as illustrated in Figure 3, the recoalescence vertex simply becomes the new gMRCA.

Recombination vertices are “added”, figuratively speaking, by deleting an edge from the graph and connecting the new vertex with both endpoints of the removed edge. The edge connected to the child vertex is the recombination vertex’s child edge, and the other is its left edge. This procedure leaves the right edge floating. Every recombination is immediately followed by the coalescence of the right edge with the graph. As it is carried out by a different algorithm than the coalescence of two dangling vertices encountered in temporal algorithms or, more trivially, when inferring a coalescent tree, we call those *recoalescence* events even though they result in an additional coalescence vertex as well. Coalescence and recombination vertices are in many regards mirror images of each other. A coalescence vertex has two child edges and one parental edge. If the child edges are ancestral for two intervals $I_{l}$ and $I_{r}$ , then so is the parental edge for $I_{l}\cup I_{r}$ . The standard recoalescence procedure begins again by deleting an edge, followed by connecting its incident vertices with the recoalescence vertex. The remaining child edge is then connected to the recombination vertex’s right edge, concluding the procedure. Recoalescence can also occur without edge deletion. In that case, the coalescence vertex becomes the new root of the graph and lacks a parental edge. It is connected downstream to the previous root and the recombination vertex. Both types of recoalescence, with and without edge deletion, are illustrated in Figure 3.

The type of a recoalescence event depends on its latitude, denoted $l_{c}$ , which itself depends on the recombination’s latitude $l_{r}$ . The root of an ARG corresponds to the sample’s most recent common ancestor (MRCA), sometimes called the *grand* MRCA (gMRCA). Its latitude is the time to the gMRCA (TgMRCA). Let $v_{r}$ and $v_{c}$ be the recombination and recoalescence vertices and $s_{r}-d_{r}$ and $s_{c}-d_{c}$ the edges deleted in the recombination-and-recoalescence (RR) steps. Algorithm 2 summarizes the RR procedure.

Algorithm 2 **Recombination-and-recoalescence.**

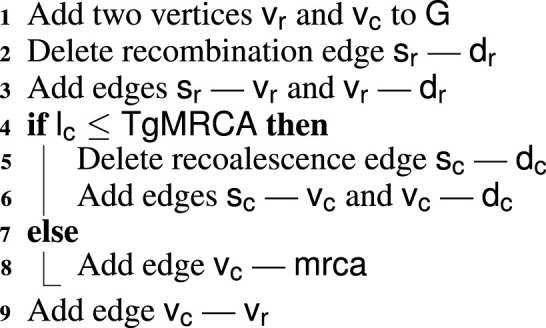

#### 2.2.1 Unrestricted recombination-and-recoalescence events

Moonshine has the capability to generate two kinds of RR events: restricted and unrestricted. Unrestricted events, the subject of this section, have a distribution designed to closely match that of the CWR. Restricted recombination events are designed to reduce the total number of mutation events on an ARG; these will be discussed at length in the next section.

**FIGURE 4:**
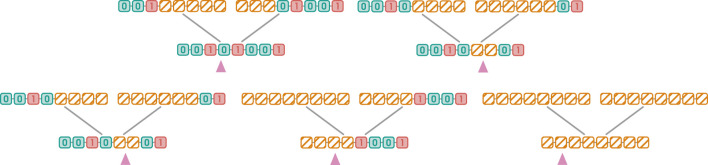
Types of recombination events. Pink arrows indicate the positions of breakpoints.

Our package exports a method for generating an arbitrary number of unrestricted recombination events. Standard theory Wiuf and Hein (1999) models the positions (on sequences) and locations (on ancestries) of recombination events as a Poisson point process (PPP). Conditional on their number, both locations and positions are distributed uniformly. When applied directly, this method has the drawback of generating sequences devoided of material ancestral for the sample at hand. Recombination events can be classified depending on whether ancestral material is present on both sides. If it is, the breakpoint can be positioned in ancestral or non-ancestral material. These are referred to as *type 1* and *type 2* events respectively and exclusively create haplotypes having ancestral material. Events positioned such that only material to their left or right side is ancestral are classified as *type 3* and *type 4* respectively. Finally, events occurring in entirely non-ancestral sequences are classified as *type 5*. Examples for each type of event are provided in Figure 4. It is desirable for an algorithm to only generate the first two types of recombination events since the other ones do not contribute to the structure of the sample. Our method follows this approach. We start by drawing a recombination edge with probability proportional to its length. The location of the event is distributed conditional on the selected edge. Then, we choose a position, also known as a *breakpoint*, uniformly on the mathematical closure of the set of intervals for which the recombination edge is ancestral. This strategy enforces the existence of ancestral material on both sides of the breakpoint and avoids recombination events of type 3, 4 and 5. Additionally, since the closure of ancestral intervals is not, in general, equal to the intervals themselves (it may contain “holes” of non-ancestral material), type 2 events are possible.

Selecting a conditional distribution for the location of recombination events on branches requires careful consideration. Theory dictates that it should be uniform. However, this results in a problematic frequency of locations close to the branches’ endpoints, contributing to the *short branches issue* discussed in Subsection 2.2.5. Consequently, we use the location-scale family associated with the Beta(2,2) distribution instead. This method preserves the symmetry of the uniform distribution while favoring locations closer to the center of branches, achieving sufficient reduction in the number of short branches to prevent numerical error.

The recoalescence process follows standard theory. The latitude is distributed as an inhomogeneous Poisson process with rate equal to the number of branches. The usual time-scale transformation strategy (see Subsection 2.2.5) is implemented. A recoalescence edge is selected uniformly, conditional on the latitude. The process of generating a single unrestricted recombination event is summarized in Algorithm 3.

Algorithm 3 **Unrestricted Recombination.**

**1** Select a recombination edge in ${\text{e}}_{r}\in E$ with probability proportional to its length
**2** Choose a breakpoint $\text{r}\in \left(r_{L},r_{U}\right)$ where $r_{L}$ and $r_{U}$ are the leftmost and rightmost positions for which e_r_ is ancestral
**3** Generate a recombination latitude l_r_ distributed as $\text{ldst}{\text{e}}_{r}+\left(\text{lsrc}{\text{e}}_{r}-\text{ldst}{\text{e}}_{r}\right)\text{b}\left(2,2\right)$ where lsrce **(e**_**r**_ **)** and ldst **(e**_**r**_ **)** are the latitudes of the highest and lowest vertex adjacent to e_r_ respectively
**4** Generate a recoalescence latitude l_c_ distributed as an inhomogeneous PPP with rate equal to the number of branches via time-scale transformation
**5** Select a recoalescence edge e_c_ uniformly among the edges at latitude l_c_
**6** Apply Algorithm 2

As a sidenote, since a function allowing users to add arbitrary recombination events to a graph is exposed, Moonshine can easily be used to simulate ancestries. We do not recommend doing so, however, especially not using the built-in types Tree and Arg, which are designed to implement ARG inference. Instances of these types store information irrelevant in a simulation context which greatly hinders performance.

#### 2.2.2 Restricted recombination-and-recoalescence events

Moonshine can be used for ARG inference due to its ability to transform an inconsistent graph into a consistent one by generating a sequence of RR events. A typical run starts with the construction of a coalescent tree and is followed by a sequential sweep made up of the following broad steps:

1. Start at the leftmost position;
2. Find the next position that is inconsistent with the sample;
3. Generate a restricted recombination event;
4. Repeat until the rightmost position is reached.

To ensure our graphs’ distribution closely resembles that of the CWR, our aim, alongside computational efficiency, is to impose as few constraints as possible on restricted recombination events. The probability distributions involved are similar to their unrestricted counterparts, except for their support, which we attempt to reduce only as much as is necessary to enforce consistency of the final ARG.

#### 2.2.3 SIMD-accelerated minimum mutation number algorithm

Step 2 requires an efficient inconsistency detection algorithm. Specifically, given a starting position, it must be able to find the closest marker to its right that mutates more than once in its current marginal tree. Furthermore, efficiency considerations demand that the set of edges on which these mutation events occur, which we refer to as *mutation edges*, is computed simultaneously. We refer to algorithms fulfilling these two requirements as *minimum mutation number* (MMN) algorithms. Mutation edge identification is at the core of Moonshine’s ARG inference procedure and it should come at no surprise that a lot of time and effort went into its optimization. It is key to enabling inference on real-world sized datasets. We begin this section by giving a general description of the procedure before delving into implementation specifics.

Our algorithm leverages the parallel introduced in Subsection 2.1 between sequences of s biallelic markers and s -vectors of GF(2) . In addition to ⊕ defined earlier, denote the multiplication on GF(2) , which is identical to the one for natural numbers, by ⊗ . Within that framework, both binary operations can be given a biological interpretation. Let $h_{1}$ and $h_{2}$ be two vectors of $\mathrm{GF}{\left(2\right)}^{s}$ and let $h=h_{1}⊕h_{2}$ where ⊕ is applied elementwise. Since the characteristic of GF(2) is 2 (i.e., 1⊕1=0 ), any non-zero element $h^{\left(0\right)}$ of h results from $h_{1}^{\left(0\right)}\neq h_{2}^{\left(0\right)}$ . This, in turn, indicates an odd number of mutations occurring between the two sequences at that marker. Assuming a mutation model disallowing back mutations, this simplifies to exactly one mutation event.

Interpretation of ⊗ is slightly more convoluted. Let v be an internal vertex and $v_{1},\ldots ,v_{m}$ the subset of leaves having v as an ancestor. Denote the corresponding sequences by $h_{v},h_{v_{1}},\ldots ,h_{v_{m}}$ . Assuming no back mutations,

$$h_{v}=h_{v_{1}}⊗\cdots ⊗h_{v_{m}}$$

where, again, ⊗ is applied elementwise. This is easily seen to be the case for a vertex v having two leaves $v_{1}$ and $v_{2}$ as children. The only way $h_{v}^{k}=1$ is if $h_{v_{1}}^{k}=h_{v_{2}}^{k}=1$ since at most one k -mutation could have occurred on $v-v_{1}$ and $v-v_{2}$ . For the same reason, if $h_{v}^{k}=0$ , then either $h_{v_{1}}^{k}=h_{v_{2}}^{k}=0$ , in which case no mutation occurred, or $h_{v_{1}}^{k}=h_{v_{2}}^{k}⊕1$ , in which case a single one occurred. This explanation can be extended to “deeper” vertices by moving upward, labelling internal vertices encountered along the way.

The correspondence between GF(2) and the evolution of sequences of biallelic markers under a model precluding back mutations can be exploited for computational efficiency. In practice, for large samples, our implementation reduces the time needed to compute mutation edges to about 15% of total execution on recent hardware, and this is without resorting to any form of multithreading. Nearly all of the time spent in the MMN algorithm is devoted to graph traversal. This level of performance is made possible by the following data structure: A sequence $h=h^{1}\cdots h^{s}$ can be associated to the number $\tilde{h}$ whose binary representation has its first s least significant digits corresponding to h . In order to make this representation unique, we assume that the other digits of $\tilde{h}$ are 0. This corresponds to

$$\tilde{h}=\overset{s}{\underset{k=1}{\sum }}2^{k}h^{k}.$$

When context is unambiguous, we shall drop the tilde and denote by h either a haplotype, the corresponding vector or its integer representation. Treating haplotypes as integers is very convenient as operations ⊕ and ⊗ described above correspond to the bitwise exclusive disjunction (XOR) and conjunction (AND). For that reason, we denote those by ⊕ and ⊗ as well. This interpretation allows for very efficient handling of sequences since these are single instructions in computer processors: we cannot make mutation finding any simpler or faster with respect to computational hardware. Given an edge $v_{1}-v_{2}$ ancestral for an interval ω , we can find all of its mutations *via* we can find all of its mutations *via* Algorithm 4:

Algorithm 4 **Mutation Events on an Edge.**

**1** Compute a ⊗ -mask $m_{\omega }$ masking markers outside ω to 0
**2** Compute $h=\left(h_{v1}⊕h_{v_{2}}\right)⊗m_{\omega }$

**3** Find the indices of set bits in h

Modern computers generally read data from memory in chunks of 64 bits and can apply elementary bitwise operations such as XOR and AND to such chunks in a single cycle. This is readily implemented and results in the concurrent assessment of 64 markers on two haplotypes on a single physical core. This is good, but we can do even better. On recent hardware, some operations can be applied to chunks larger than 64 bits, a capability enabled by so-called single instruction, multiple data (SIMD) instructions. At the upper end of the spectrum are processors supporting the AVX-512 instruction set, which can operate on 512 bits simultaneously. Such operations are said to be “vectorized” in reference to them being applied to multiple 64 bits “scalars” at once. While not all regular CPU operations have a vectorized counterpart, integer AND and XOR, the only two operations required for mutation detection, are part of the AVX-512F extension available on any AVX-512 compliant processor.

**FIGURE 5:**
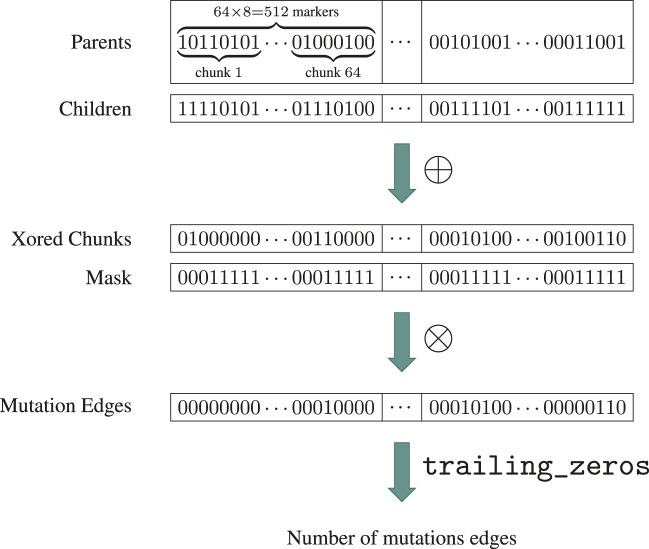
Schema of the MMN algorithm on an AVX-512 compliant architecture. After the ancestral edges for the interval of interest ω have been listed, the haplotypes of their parent and child vertices are divided into chunks of 8 markers, which are stored in two separate arrays: the first chunk in the “Parents” array corresponds to the parent of the first edge, the second chunk to the parent of the second edge, and so on. The “Children” array is constructed similarly. Each matching pair of cells from both arrays is then xored in a single operation, resulting in the “Xored Chunks” array. The bitwise conjunction between each chunk and the mask $m_{\omega }$ is then computed in a similar fashion. Note that the “Mask” array exists for illustrative purposes only and does not need to be instantiated in practice. The number of mutation edges is obtained by repeated bitshift and application of trailing_zeros on “Mutation Edges”. The mutation edges can be obtained simultaneously *via* the indices of set bits.

That being said, it would generally not be very efficient to compare chunks of 512 markers from 2 different haplotypes simultaneously, as the next mutating marker is likely near the last recombination breakpoint. Instead, we process haplotypes in chunks of 8 markers. On an AVX-512 enabled architecture, this results in up to 64 haplotypes being compared at 8 positions concurrently without multithreading as illustrated in Figure 5. In fact, this is so efficient that tested multithreaded versions were actually slower than the single-threaded one. Our algorithm is implemented in Moonshine using SIMD.jl (Schnetter et al., 2025) which ensures portability across architectures. A custom fallback is implemented to allow use on hardware that does not support AVX.

Computing the number of mutations by batches of 8 markers naturally leads to optimizations of the graph traversal procedure. The main one is based on the observation that, assuming no back mutation, a given marker cannot have the derived allele for a haplotype if one of its descendants has the wild allele. This is the biological version of 0 being the absorbing element of ⊗ . By performing traversal in a bottom-up fashion starting at the leaves, we allow for early termination when encountering a vertex associated with a chunk of zeros. The probability of early termination increases as the size of the chunk of markers decreases, further justifying our decision to work with byte-sized chunks. The complete MMN procedure is given in Algorithm 5. Storing traversed edges and associated chunks before applying Algorithm 4 (line 9) is wasteful but necessary to exploit SIMD parallelism. Line 22 assumes that markers are encoded right-to-left within chunks (e.g., the sequence 111,000 is stored as 000111). On modern architectures, trailing_zeros essentially correspond to a single processor instruction on modern hardware and is consequently very fast. idx_to_pos (line 27) is the function that computes the position of a marker from its index; see Subsection 2.2.10 for details. The chunk size of 1 byte (8 bits), assumed throughout Algorithm 5, is a compile-time constant for performance reasons. Although we believe it to be the best choice in most circumstances, it can easily be configured *via* Julia’s preferences mechanism.

Algorithm 5 **MMN.**

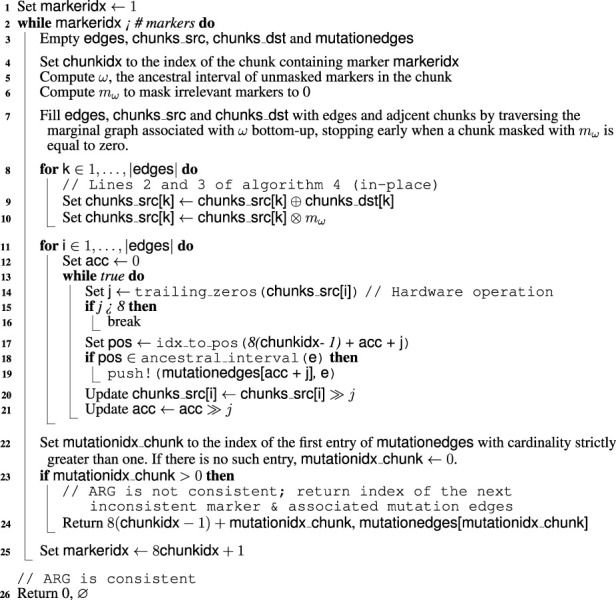

#### 2.2.4 Breakpoint

Once an inconsistent marker has been found, a sequence of RR events is generated in a way that reduces the local number of mutations down to one. The RR procedure itself is discussed in details in Subsection 2.2.5. For now, we are concerned with the distribution of breakpoints.

Standard theory models the occurrence of recombination events as a Poisson process, which leads to an elementary algorithm for sequential simulators revolving around sampling exponential interarrival times. Things are different when inferring ARGs. Assuming a strong mutation model such as the ISM greatly restricts the number and position of breakpoints. Let b and m be the positions of the last breakpoint and the next inconsistent marker, respectively. Since, under the ISM, some events must be positioned in this interval, it would not make sense for the distance between b and the next breakpoint to follow an exponential distribution. A standard result about PPPs states that events are uniformly distributed in an interval conditional on the number of events in that interval. From this point of view, the correct way of choosing breakpoints in $\left.\left[b,m\right.\right)$ would be to first draw a Poisson-distributed number of events and then position them uniformly in $\left.\left[b,m\right.\right)$ . Unfortunately, generating a sequence of events that would yield a tree consistent with the marker at m having a predetermined length is prohibitively difficult unless we are ready to either engage in additional time-consuming computation or exclusively locate recombination events on *derived edges*, that is those edges whose downstream vertex (the one closer to the leaves) has the derived allele at the focal marker. As described in Subsection 2.2.5, the maximum number of mutations eliminated by a recombination event occurring on a *wild edge*, that is, an edge whose downstream incident vertex is associated with the wild allele for the marker under consideration, is the number of mutations in the marker’s marginal tree minus one. In other words, depending on the ARG’s topology, a constrained RR event might remove anywhere from a single mutation to all of them.

An additional complication is that while choosing breakpoints in $\left.\left[b,m\right.\right)$ leads to a reduction in the number of mutations for the marker at m assuming correct locations for RR events, there is no guarantee that doing so will not create mutation edges for markers to the left of m . Since we are inferring ARGs from left to right, increasing the number of mutation edges for markers to the right of m is not a problem. It would generally be desirable for that number to go down, but designing a scalable algorithm to achieve that goal would be challenging, since mutation edges associated with every marker to the right of m would need to be computed for multiple breakpoints in $\left.\left[b,m\right.\right)$ and, for each breakpoint, multiple sequences of RR events. In any case, additional mutation edges created for markers to the right will be dealt with in subsequent iterations. Creating mutations for markers that have already been processed is more problematic. While we could backtrack to deal with those, this would negatively affect performance and necessitate generating additional events. Instead, Moonshine imposes additional constraints on breakpoints’ distribution. Let $m^{\prime }$ be the position of the first marker to the left of m . An event positioned in $\left.\left[m^{\prime },m\right.\right)$ cannot create mutation edges for markers to the left since any associated edge is not ancestral for those markers.

Restricting the support of breakpoints to the interval between the next inconsistent marker and the one directly to its left is efficient but overly prohibitive. In addition to reducing the support of breakpoints for no reason other than ease of implementation, it diminishes our algorithm’s ability to generate type 2 recombination events. Algorithms based on a first-order Markov approximation of the recombination process, such as SMC and SMC’, take a rather drastic approach by simply ignoring any event that might have happened anywhere except on the current marginal tree. Other algorithms allow recoalescence events to happen further along the sequences while still restricting the position of recombination events. The main selling points of these algorithms are their computational efficiency and relative simplicity. However, since recombination events are precluded from happening in non-ancestral material, they are inherently incapable of generating type 2 recombinations. In order to simulate these, a sequential algorithm must be able to go back in space, so to speak, while simulating the recombination process: some events must happen to the left of the preceding one. This class includes algorithms such as those implemented by SC and, by extension, SC-sample. Their strategy is to generate a recombination event on either the coalescence branch of the previous RR event or one located upstream. This process is repeated until recoalescence with the current marginal tree occurs. ARGinfer is another algorithm able to produce type 2 recombination. Being an MCMC-based, it does so *via* two proposals: “adding a new recombination to a lineage” and “resampling the breakpoint of a recombination event” (Mahmoudi et al., 2022).

These algorithms have different approaches to type 2 events. One approach, used by SC, aims to stay closer to the coalescent process by generating recombinations according to a complex distribution. The other, used by ARGinfer, uses a less sophisticated distribution and relies on the properties of its underlying MCMC sampler for statistical correctness. It is apparently able to propose a move faster than SC can generate a recombination event. However, many of those moves may be rejected before one is finally accepted, while every event generated by SC is actually integrated into the ancestry. Both approaches have their merits, and it would be risky to make a unilateral statement as to which is the best.

Our package shares many characteristics of SC and ARGinfer, but its approach to type 2 events is different. Since we are processing haplotypes from left to right and only consider events resulting in a reduction of the marginal number of mutation edges, breakpoints associated with type 2 events have to be positioned to the left of not only m but of $m^{\prime }$ as well. This begs the question: what are the conditions under which a recombination event positioned at $b\leq b^{\prime }<m^{\prime }$ does not increase the number of mutation edges in $\left.\left[b,m\right.\right)$ ? Let $e_{r}$ , $e_{c}$ be the recombination and recoalescence edges respectively, $v_{r}$ , $v_{c}$ the associated child vertices, and $h_{v_{r}}^{k}$ , $h_{v_{c}}^{k}$ the status of the k ^th^ marker of the associated haplotypes. An RR event positioned at breakpoint $b^{\prime }$ will not result in the creation of additional mutation edges in $\left.\left[b,m\right.\right)$ as long as for every marker k in $\left[b^{\prime },m\right)$ , one of the following conditions is met:

1.
$h_{v_{r}}^{k}=h_{v_{c}}^{k}$ ;
2.
$e_{r}$ is not ancestral for marker k ;
3.
$e_{c}$ is not ancestral for marker k and the status of the first ancestral vertex upstream $v_{c}$ is $h_{v_{r}}^{k}$ .

Among these conditions, item 2 allows for type 2 events. Since we are working from left to right, support for those breakpoints comes with the potential for type 4 events as well. Consequently, they must be explicitly discarded.

Assuming the lower limit of the support for the i ^th^ breakpoint $b_{i}^{\prime }$ is known, we need to decide on a distribution for the breakpoint itself. Since the support is bounded, the two most natural choices are the truncated exponential and uniform distributions. The main advantage of the former is its resemblance to the untruncated exponential distribution, which is the mathematically correct choice assuming a PPP and an unknown number of recombination events in $\left.\left[b,m\right.\right)$ . For minimum departure from this model, the i ^th^ breakpoint $b_{i}$ would be supported on $\left[\max\left\{b_{i}^{\prime },b_{i-1}\right\},m\right)$ where $b_{0}=0$ . In addition to precluding type 2 events, this distribution has the drawback of being difficult to deal with from a numerical standpoint. Each draw reduces the support of the next, making standard approaches to random generation such as rejection sampling and inverse transform less efficient and/or more prone to instability due to divisions and the use of transcendental functions. Consequently, Moonshine uses the simpler uniform distribution. Each breakpoint is supported on the full interval $\left[b_{i}^{\prime },m\right)$ which avoids numerical instabilities and allows for type 2 events. This is akin to trying to stay as close as possible to the PPP distribution assuming (erroneously) a known number of events.

#### 2.2.5 Recombination and recoalescence

When processing sequences from left to right, the procedure described in the previous section allows to efficiently find the next site incompatible with the current ARG, that is, the site with two or more mutation edges. It is straightforward to keep track of these by storing them in a list, for instance. The next step is then to add recombination events to the ARG in a way that renders it consistent with the focal site.

**FIGURE 6:**
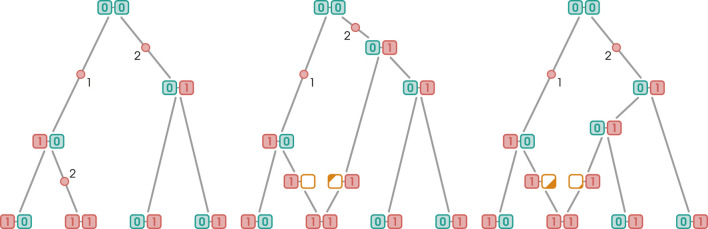
Derived recombination followed by a recoalescence event on a derived edge. Original graph is represented in Figure 6. Figure 6 are example of the two possible types of recoalescence events.

Let M be the set of mutation edges. The coalescence of two elements of M results in a net diminution of the number of mutation edges by 1. The reason is as follows: since the two coalescing edges have the same allele at the focal site (namely, the derived one), neither will be a mutation edge anymore after coalescence. However, one edge upstream of the coalescence vertex will ultimately coalesce with a wild edge and therefore become a new mutation edge, resulting in the aforementioned net diminution. To conclude the argument, note that it is not possible for a coalescence event to decrease the number of mutation edges by more than 1. For that to be possible, one of the coalescing edges would need to have a sibling that is itself a mutation edge. However, in that case, the coalescing edge would not be a mutation edge. We call recombination events located on a derived edge and the recoalescence event that follows *derived RR event*. A configuration resulting from such a pair of events is given in Figure 6. The previous argument actually entails an even stronger conclusion: there is no requirement for the edge on which recoalescence occurs to be a mutation branch. As illustrated in Figure 6, merely being derived is sufficient.

**FIGURE 7:**
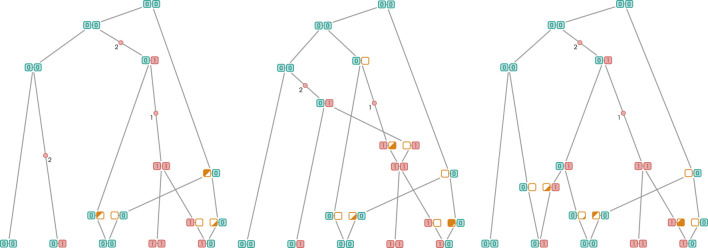
Derived recombination event located on an edge downstream of a mutation edge. Reduction in the number of mutation is achieved since the recombination edge has no sibling marginally.

The type of derived events we just described is said to be *constrained* because their locations and positions are supported on a subset of the ARG branches and sequences, respectively. Ideally, this subset should encompass as many branches as possible, making the distribution closer to the true (i.e., PPP) one. It is in fact possible to extend the set of candidate branches without compromising the reduction of the number of mutations. For one thing, recombination events do not have to be located on a mutation edge. Let u−v be such an edge with v being the downstream vertex. Since v is derived, so is every vertex located downstream in the current marginal tree. Let $E_{v}$ be the set of edges downstream of v in that tree. The number of mutations can be reduced by one by taking a subset of $E_{v}$ separating v from the ARG’s leaves and generating an RR event on each edge, provided that each recoalescence events is located on derived edges outside $E_{v}$ . If we want to avoid inflating the number of recombination events, we can impose the additional restriction $|E_{v}|=1$ and generate a recombination event on u−v if no such edge separator satisfies this constraint. This is easily achieved in practice by looking for sequences

$$u-v,v-w_{1},w_{1}-w_{2},\ldots$$

where each edge is marginally without sibling. In particular, this is the case if $v,w_{1},w_{2},\ldots$ are recombination vertices, although it is not a necessary condition, as illustrated by Figure 7. Considering these edges as possible locations for recombination events allows for a more even distribution, which, in addition to making the approximation to the true distribution, contributes to the elimination of numerical errors associated with a large number of such events relative to the total branch length. Sequential algorithms come with something of a numerical curse: as the ARG grows, so does the number of short branches resulting from events located close to a branch’s incident vertices. Soon enough, numerical instabilities arise when a short mutation edge is encountered. Fortunately, as we have seen earlier, the remedy is simple to implement.

We go even further by applying a similar analysis to the location of recoalescence events. As noted by Wang et al. (2014), these do not need to be supported on derived edges. A recoalescence with a non-ancestral edge downstream of a derived edge does not increase the number of mutations, a scenario illustrated in Figure 7. Since it is impossible for a wild edge to lead to a derived one, the set of admissible locations for recombination events can be described in rather simple terms: coalescence of a derived edge with a mutation edge or any of its downstream edges does not increase the number of mutations.

It should be clear by now that we can make any position consistent by generating at most |M|−1 derived recombination events. This is good, but we can actually do better, at least some of the time. We refer to recombination events located on a wild edge and the subsequent recoalescence event as *wild RR event*. Despite not occurring on a mutation edge, a wild recombination event can effectively reduce the number of mutations when the following two conditions are met:

1. The recombination edge’s brother is a mutation edge;
2. Their uncle is a mutation edge.

**FIGURE 8:**
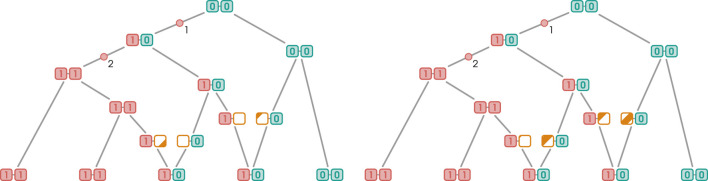
Wild RR event leading to a reduction in the total number of mutations with recoalescence on non-ancestral edge.

In such a configuration, the recombination event turns both derived vertices, namely, the sibling and uncle of the recombination vertex, into siblings with respect to the current marginal tree, reducing the number of mutations by one. As with the derived case, the wild recombination event does not need to occur on the sibling of the mutation edge; the number of mutations will be reduced as long as it is located downstream somewhere along a “chain” of edges without marginal siblings. Just like in the derived case, coalescence can occur with non-ancestral edges as long as they lead to a wild branch; this is illustrated in Figure 8. Failing to meet this condition would result in the creation of a new mutation event.

**FIGURE 9:**
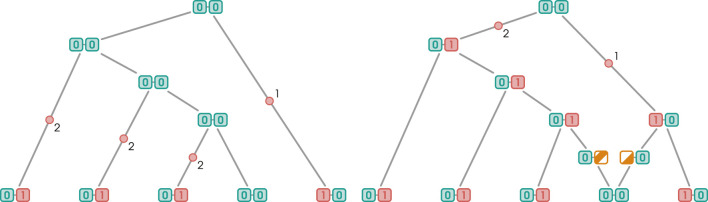
A single recombination event leads to the elimination of multiple mutations. This is only possible when the recombination event occurs on a wild branch adjacent to a series of “derived cousins”, a configuration illustrated in Figure 9.

When it comes to the number of mutations eliminated, wild RR events clearly have the upper hand. As we just discussed, a wild recombination event flips the allelic state of the mutation edge’s parental vertex to derived. Consequently, a reduction in the number of mutations can propagate upward as long as the uncles encountered along the way in the current marginal tree are also mutation edges. In fact, given an appropriate topology, a single mutation event can reduce an arbitrary number of mutations to a single one. A scenario where a single coalescence/recombination event leads to the elimination of two mutations is illustrated in Figure 9. On the contrary, as discussed before, the number of mutations eliminated by a derived RR event is limited to one. This is because the mutation is eliminated by the recoalescence rather than the recombination event. The state of the marker of the parental vertex of the mutation edge on which the recombination event occurred remains unchanged, inhibiting the propagation phenomenon observed in the wild case.

That being said, although Moonshine has the capability of inferring ARGs by generating mutation reducing events exclusively, it does not actively seek to maximize mutation reduction locally. It does not, for instance, give priority to wild events over derived ones. As our main objective is to remain close to the CWR, only branch length is taken into consideration when choosing the location of a recombination event. Each edge on which a recombination event would result in a reduction in the number of mutations for the current marker has a probability of being chosen proportional to the difference in latitude of its incident vertices, a strategy described earlier as “constrained”. For reasons discussed earlier, the event’s location on the branch is not selected uniformly but rather assumed to be distributed as the same location-scale family associated with the Beta distribution as the one used for the unconstrained case.

Determining the location of a derived recoalescence event is fairly straightforward. According to standard theory (Wiuf and Hein, 1999), coalescence occurs at a unit rate with each admissible edge. We simply need to list all available locations and pick one uniformly at random. The set of possible recoalescence edges $E_{R}$ is readily established and does not require graph traversal. The minimum latitude is either that of the recombination event or the smallest latitude among the destination vertices of $E_{R}$ , whichever is greater. Each edge in $E_{R}$ is associated with a probability proportional to its length minus any section outside of admissible latitudes. Once the recoalescence branch is determined, a location is selected as usual on its admissible portion.

Simulating the location of a wild recoalescence event is more involved due to the semi-infinite nature of its support. Its latitude is distributed as the time of the first event of a non-homogeneous Poisson process. A common approach in one-dimensional scenarios such as ours is the *time-scale transformation* method, which is a form of inverse transform sampling and requires computation of the inverse of the integrated intensity function, also known as the *cumulative intensity function*. The main issue stems from our decision not to track the number of live edges by latitude. This improves general performance and reduces memory usage, but makes evaluation of the intensity function extremely time-consuming, as it requires graph traversal. We tackle this issue using numerical integration. Although somewhat variable, the intensity function is piecewise constant and therefore a very good candidate for quadrature. We use a logarithmic grid of latitudes to account for the generally decreasing complexity of the ARG topology as height increases. The intensity function is evaluated at quadrature nodes in a single partial traversal of the ARG, reducing overhead to the minimum. By default, our algorithm uses a grid of compile-time constant size 25, but this number can be tuned to balance precision with performance using the preference mechanism.

#### 2.2.6 Multiple crossing over

**FIGURE 10:**
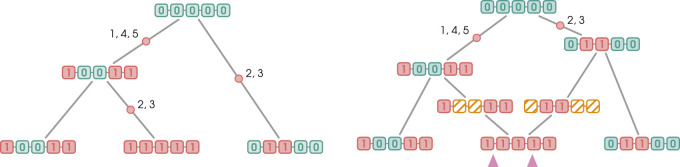
Multiple crossover events. Breakpoints are indicated by pink arrows.

In addition to RR events, Moonshine can generate multiple crossing over events constrained to reduce the number of mutations. These events arise in the following scenario: assume that the branch selected to undergo recombination is the right parental edge of a recombination vertex. Assume further that a recoalescence with the sibling of the other parental edge’s branch would reduce the number of mutations. When both of these conditions are met, RR events are unnecessary; as illustrated by Figure 10, the same effect can be achieved by modifying the ancestral intervals of the parental edges of the recombination vertex. For this to work, it is necessary to track the partition induced by recombination events. In an ARG, every recombination vertex is associated with two sets of intervals, one for each parental edge. We call these sets *recombination masks* and denote the mask associated with recombination k by $m_{k}=\left\{m_{k}^{l},m_{k}^{r}\right\}$ . Initially, before any MCO event involving a specific recombination vertex, the mask is rather simple. Let b be the position of the associated recombination event.

$$\begin{array}{ll} m_{k}^{l} & =\left[0,b\right)\\ m_{k}^{r} & =\left[b,\infty \right). \end{array}$$

A MCO event at position $b^{\prime }>b$ transforms those sets as follows:

$$\begin{array}{ll} m_{k}^{l} & =\left[0,b\right)\cup \left[b^{\prime },\infty \right)\\ m_{k}^{r} & =\left[b,\infty \right)\cap \left[0,b^{\prime }\right)=\left[b,b^{\prime }\right). \end{array}$$

A subsequent event at position $b^{\prime \prime }>b^{\prime }$ would yield

$$\begin{array}{ll} m_{k}^{l} & =\left[0,b\right)\cup \left(\left[b^{\prime },\infty \right)\cap \left[0,b^{\prime \prime }\right)\right)=\left[0,b\right)\cup \left[b^{\prime },b^{\prime \prime }\right)\\ m_{k}^{r} & =\left[b,b^{\prime }\right)\cup \left[b^{\prime \prime },\infty \right). \end{array}$$

In general, the correct mask can be computed by intersecting the rightmost interval (with respect to the right endpoint) with $\left[0,b^{\prime }\right)$ and taking the union with the other interval and $\left.\left[b^{\prime },\infty \right.\right)$ . Although explicit storage is not necessary, coalescence vertices can be thought of as being associated with the mask $\left.\left[0,\infty \right.\right)$ . Recombination masks are used to compute the ancestral intervals of parental edges in the following way: an edge’s ancestral interval is equal to the intersection of its recombination mask with the union of its children’s intervals.

Conditions under which a MCO event can occur are very restrictive. Consequently, we expect detection to be underpowered. This might be improved in the future, for example, by allowing the user to bias the procedure in favour of these events.

#### 2.2.7 ARG update

RR and MCO events both modify sequences and ancestral intervals associated with vertices and edges upstream. The affected elements must be updated immediately to ensure the soundness of the remainder of the procedure, which can be achieved by traversing the ARG from the recombination and recoalescence edges toward the root. Sequences and intervals updates are, however, computationally demanding. Our first, rather naive implementation was a major bottleneck of the constrained recombination algorithm. To make it as efficient as possible, we limit the update procedure to the elements affected by the event. Recombination events are mostly local, meaning they can only affect vertices and edges located upstream. Consequently, any element not upstream of either the recombination or recoalescence edge can be ignored when updating. In fact, an element must be located upstream of a modified element to be modified itself. This means that the number of updates can be further reduced by keeping track of the state of every vertex and edge before they are updated and stopping when a match between original and updated versions is detected. The algorithm terminates early if both the sequence and the ancestral interval associated with an edge are left unchanged.

The early termination strategy described above dramatically decreases the time dedicated to ARG update. Indeed, coalescences with vertices left untouched limit the spread of changes, often well below the root. It might be conceived that the additional costs associated with storing information about elements before updating them would outweigh the benefits of reducing the number of updated elements. It turns out that a very significant number of recombination events are very local in nature, to such a degree that we have yet to find the point of diminishing return for reasonably large samples. That is not to say, however, that the procedure cannot be improved further. We were able to squeeze even more performance out of it through hashing. Instead of making a copy of the current sequence and ancestral interval before update, we simply compute a hash value for each of those, which we promptly hash together. The procedure is terminated early if the hash of the updated sequence-ancestral interval pair is equal to the original. Since hash functions are not injective, there is a possibility that different pairs may have the same hash, an event known as a *collision*. Fortunately, this has not been a problem in practice. We use a universal hash function from the 64-bit NH family Black et al. (1999) which have a pairwise collision probability of at most $2^{-64}$ . To put any doubt to rest, the method validate can be applied to the final product of the ARG inference process to ensure that it is exempt, among other things, of the inconsistencies that would emerge from collisions. Future versions will allow users to select from multiple hash functions within the 64-bit NH family to mitigate the effects of a collision. The complete procedure is presented in Algorithm 6. Note that since recombination vertices have a unique child, their associated sequence can be a reference to their child’s. Consequently, line 9 can be performed without additional allocation, dramatically cutting down on memory usage. The early termination strategy is implemented by line 26.

Algorithm 6 **ARG Update.**

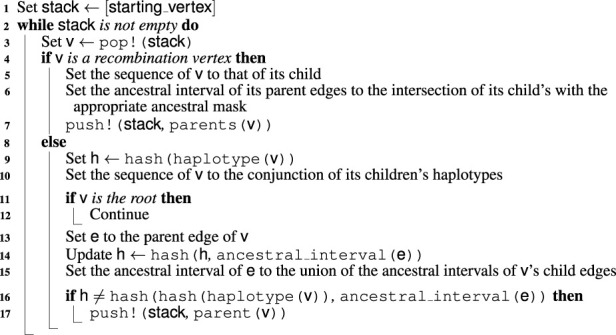

#### 2.2.8 Technical considerations

##### 2.2.8.1 Markov approximation

**FIGURE 11:**
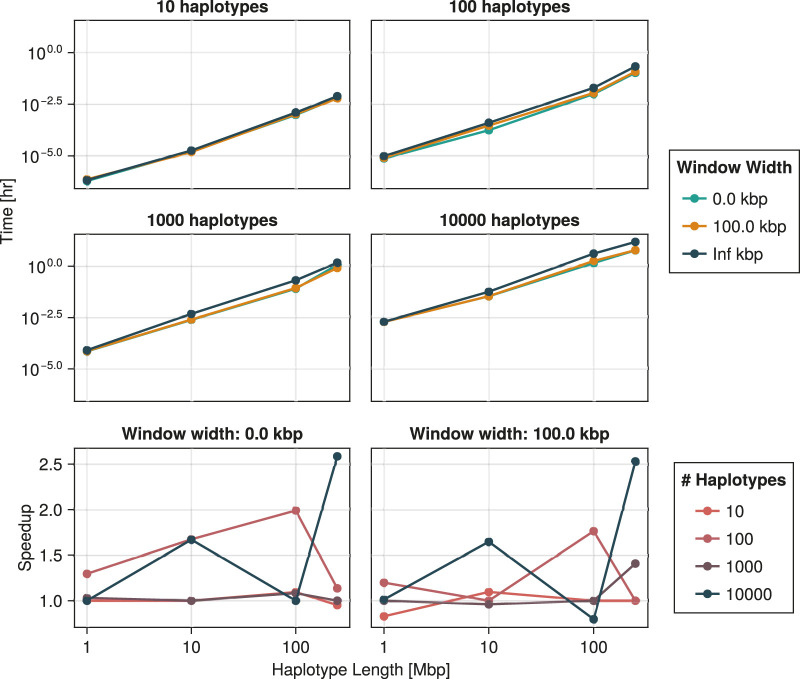
Time required to construct a single consistent ARG as a function of the number of haplotypes and haplotype length, including time spent inferring the initial tree. The speedup provided by two window widths with respect to an infinite window is depicted in the bottom two panels. Technical details are discussed in Section 3.

The procedure described in this section is exact in the following sense: at any step, any edge of the ARG can undergo recombination or recoalescence subject only to the mutation number reduction constraint. It is more similar to the original Wiuf-Hein sequential algorithm than any of its Markovian approximations, such as the SMC or SMC’. This makes ARGs more realistic, but it comes with a performance penalty: as RR events are incorporated into the graph, the number of edges that need to be considered for the next events increases. Consequently, we should expect the number of operations performed by our algorithm to grow as it progresses along sequences. One way to alleviate this computational burden is to make the recombination process artificially Markovian. The procedure we just presented allows for such an approximation, although it was omitted from the description for simplicity. Both restricted and unrestricted RR events may be executed inside a window moving across the sequences, the width of which can be selected by the user. A width of 0 corresponds to a first-order Markovian approximation akin to the SMC, while an infinite width means no approximation at all. In general, assuming distances in base pairs (bp), specifying a width of w for an RR event occurring at position p has the effect of excluding any edge e such that

$$\text{ancestral\_interval}\left(e\right)\cap \left[p-w,p+w\right]=\emptyset .$$

from the set of candidate recombination and recoalescence edges. We emphasize that the window is *centered* on p , as our algorithm is designed for the general task of rendering ARGs consistent rather than building them from a tree in a left-to-right sweep. Moreover, when generating the recoalescence latitude of an unconstrained event, ignored edges are not taken into account for the computation of the rate of the recombination latitude. Figure 11 shows that the performance impact of reducing the window’s width is significant. For some combinations of sample size and haplotype length, a window width of 0 can lead to over twice as fast computation. Interestingly, the speedup is similar for a width of 100 kbp, suggesting that even relatively modest approximations can lead to substantial reduction in computational resources. Irrespective of window size, Figure 11 suggests that computation time is exponential in both the number of sampled haplotypes and markers.

**FIGURE 12:**
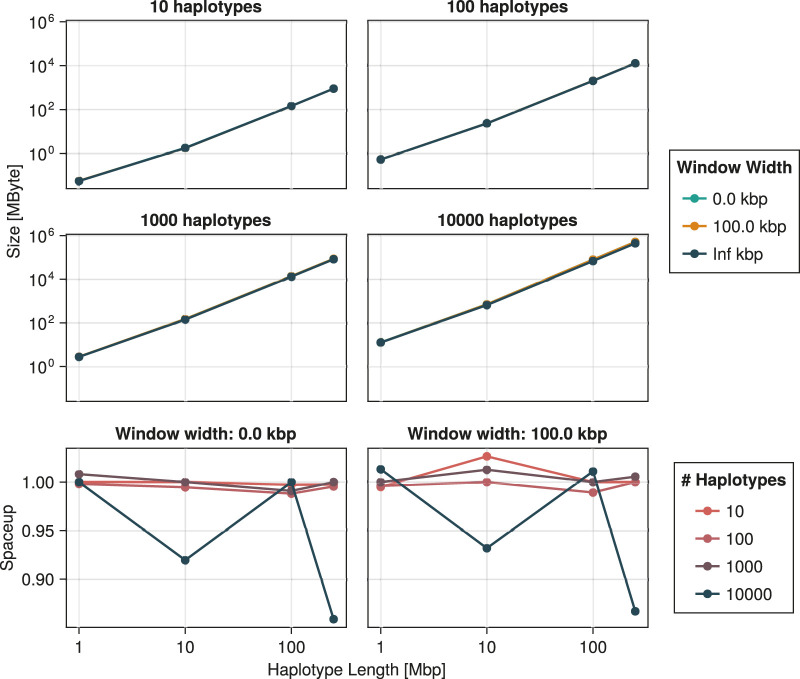
Size of the object containing the inferred ancestral recombination graph as a function of the number of haplotypes and haplotype length. The spaceup, plotted in the bottom two panels, is the ratio of the size for infinite window width *versus* that for the indicated width. Technical details are discussed in Section 3. We emphasize that the total amount of memory required by the procedure exceeds quantities reported here.

**FIGURE 13:**
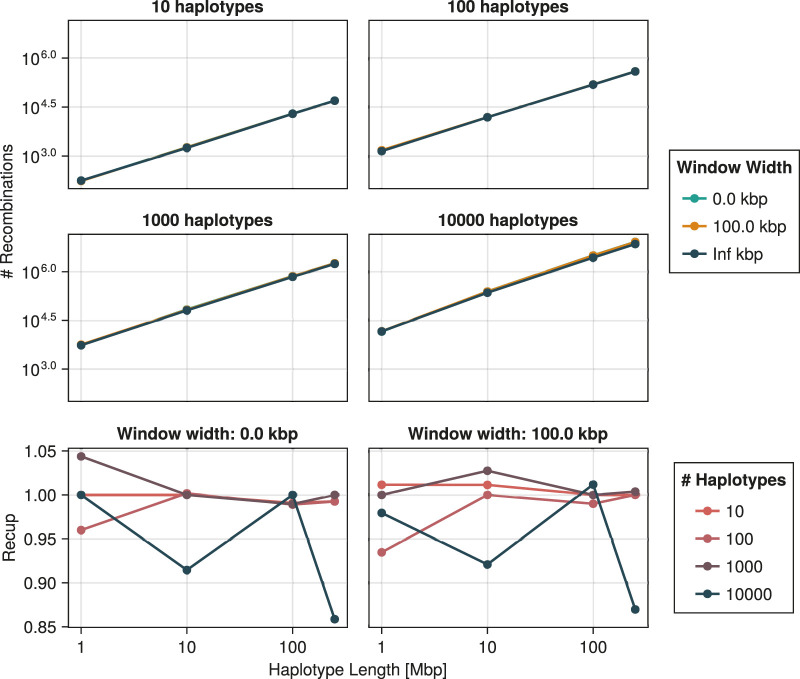
Number of recombination events generated by the ARG inference procedure as a function of the number of haplotypes and haplotype length. The recup, plotted in the bottom two panels, is the ratio of that number for an infinite window *versus* that for the indicated width. Technical details are discussed in Section 3.

Similarly, Figure 12 suggests exponential growth in memory usage. It appears, however, that reducing the window size increases the size of the resulting ARG. This is largely explained by the resulting increase in the number of recombination events. As shown in Figure 13, this number tends to increase as the window width diminishes. Part of this phenomenon might be explained by increased flexibility. Larger window sizes increase the number of candidate edges for recombination and recoalescence events, allowing for constrained coalescence of more similar haplotypes and, ultimately, more parsimonious ancestries.

#### 2.2.9 Markers and positions

In addition to the ARG update procedure described at the beginning of this section, another major bottleneck in our method was, somewhat unexpectedly, a function called pos_to_idx that returns the index of a marker, given its position. It is the inverse of another function called idx_to_pos, which is designed solely to return the position of a given marker, indexed from the leftmost one. The term “inverse” in that context is used loosely, as multiple positions are associated with a given marker. For that reason, we define pos_to_idx formally as a pseudoinverse of idx_to_pos:

$$\text{pos\_to\_idx}\left(p\right)=\sup\left\{i\in 1,\ldots ,s:\text{idx\_to\_pos}\left(i\right)\leq p\right\}.$$

Since it is often necessary to mask sequences with respect to an ancestral interval, this function is used extensively within Moonshine’s recoalescence and ARG update procedures. The endpoints of any given interval rarely correspond to the position of a marker. This is compounded by the fact that what we refer to as “ancestral interval” is, in reality, the union of multiple disjoint intervals. Overall, a simple linear search is insufficient; a more efficient approach is called for. Binary search yields considerable gains, but we can do better. Memoization is a standard strategy for solving similar problems, but it is detrimental to our use case. The cost of computing idx_to_pos *via* binary search is small, even in comparison with a lookup in a hash table. Besides, positions are encoded as double-precision floating-point values, which limit their usefulness as keys of an associative structure. Perhaps it would be possible to use locality-sensitive hashing to circumvent this issue. We did not explore this avenue, though, because the approach currently implemented, although relatively simple, proved satisfactory. It is essentially a slightly optimized version of interpolation-sequential search (Gonnet and Rogers, 1977). We take advantage of the fact that the markers’ position vector is not modified by ARG inference. Instead of directly computing the inverse of idx_to_pos, we do so on a first-order approximation obtained by least-squares fitting, which is straightforward. Even though computing such an approximation is time-consuming relative to an iteration of bisection search, it only has to be done once, making the overhead negligible. Pseudocode for pos_to_idx is given in Algorithm 7.

Algorithm 7 **pos_to_idx.**

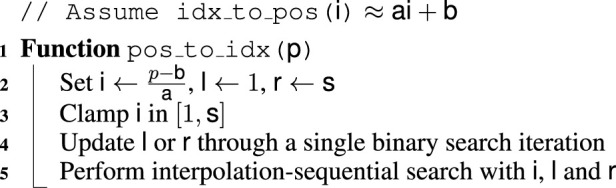

While Algorithm 7 is relatively simple, it takes advantage of three features of the positions vector: its static nature, its monotonicity, and the approximate regularity of the distance between markers. A more efficient procedure could likely be designed, but Algorithm 7 is at least efficient enough to eliminate the pos_to_idx bottleneck.

### 2.2.10 Memory allocation

Considerable effort has been devoted to using memory as efficiently as possible in the computationally demanding sections of Moonshine. In addition to the obvious benefits in terms of reduced memory requirements, we found dynamic allocation to be a major performance bottleneck in itself. Although Julia is a high-level programming language, fine-grained memory management is straightforward. It is entirely possible to manage allocations directly. Part of the C standard library, comprising functions such as malloc, calloc and free, is exposed to the user *via* the Libc module included in Julia’s standard library. Our approach, however, is slightly higher level: we make extensive use of slab allocation (Bonwick, 1994) through Bumper.jl (Protter et al., 2025). This package allows us to handle raw pointers when needed while efficiently managing the underlying memory pool automatically. This allows for nearly effortless tight memory management and dramatically reduces the number of allocations needed in core methods. Performance-critical methods accept the buffer keyword argument through which the caller can pass a Bumper.jl buffer. This is how the memory is allocated by the build! method for inferring ARG for instance. The same buffer can be passed around to different function calls for maximum memory usage efficiency. This also makes it extremely easy to seamlessly integrate Moonshine methods in a workflow that already takes advantage of Bumper.jl facilities.

### 2.2.11 Complete algorithm

The complete ARG inference procedure is given in Algorithm 8.

Algorithm 8 **ARG Inference.**

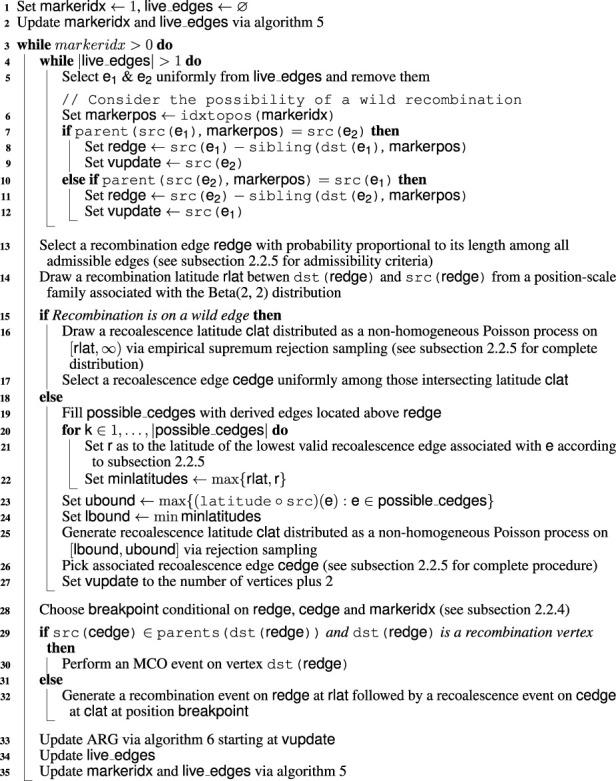

### 2.3 Comparison with other methods

#### 2.3.1 Number of recombination events

**FIGURE 14:**
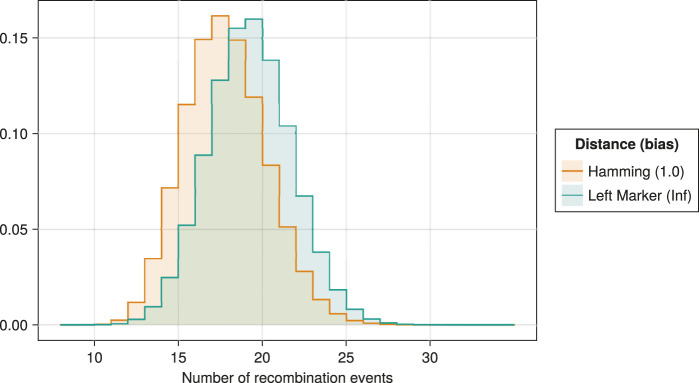
Distribution of the number of recombination events on ARGs inferred for the Kreitman dataset. The data are split into histograms based on the distance used to construct the initial coalescent tree.

To study the distribution of the number of recombination events generated by Moonshine, we inferred two million ancestral recombination graphs for the well-known Kreitman dataset Kreitman (1983). As in Wong et al. (2024), we followed the stdpopsim catalog Adrion et al. (2020); Lauterbur et al. (2023) and assumed a per-marker mutation rate and effective population size of $5.49^{-9}$ and 1720600, respectively. Half of these ARGs were based on trees constructed with the Hamming distance while the other half used the left marker distance. In both cases, the bias term $c_{0}=1$ . Distributions are illustrated in Figure 14.

The Hamming distance led to slightly more parsimonious ancestries. On average, they contained 17.43 (SD 2.52) recombination events *versus* 18.86 (SD 2.5) for the left marker-based counterpart. Additionally, the most parsimonious ancestry inferred with the Hamming distance contained 8 recombination events, just one above the theoretical minimum (assuming no recurrent mutation). This number was 9 for the left-marker-based ancestry. In both cases, the maximum number of recombination events inferred was 34.

We can compare these results with those obtained by Wong et al. (2024). Most of the ARGs inferred by Moonshine were less parsimonious than the ancestry produced by tsinfer, which contained 11 breakpoints. Relate Speidel et al. (2019), which allows for some recurrent mutations attributable to data error, is consistently more parsimonious with only 2 breakpoints. Note that neither of these two methods explicitly infers recombination events. ARGWeaver was able to construct an ARG with 37 recombination events using the SMC’ model, more than any ARG produced by our package. Finally, Moonshine is not as parsimonious as KwARG, which is able to reach the theoretical minimum. Note that KwARG uses a heuristic specifically designed for parsimony.

Thao and Vinh (2019) provides additional comparisons. It introduces GAMARG which, similar to KwARG, infers ARGs *via* a parsimony heuristic and is able to reach the theoretical minimum. In addition, the paper reports a numerical experiment in which 1,000 ancestries were inferred by ARG4WG, REARG Phuong Thao and Vinh (2017) and Margarita. The first two attained a minimum of 10 events while the last one was on par with Moonshine with 8.

#### 2.3.2 Coalescence times and topology accuracy

To assess the quality of the ancestries reconstructed by Moonshine, we simulated a topology for a sample of 100 genetic sequences of length $10^{5}$ , a recombination rate of $\rho =10^{-8}$ and an effective population size of $10^{4}$ . We then generated mutations on top of this topology according to the ISM using rates μ∈{ρ/2,ρ,2ρ} yielding three ARGs with 119, 203 and 408 polymorphic sites, respectively. For each of these, we inferred 200 ancestries from the resulting haplotypes using the Hamming distance for the initial tree and an infinite window width. For comparison, we reconstructed ARGs from the same sample using SINGER and Relate.

Relate outputs a single ancestry and is straightforward to use. Conversion to tskit’s TreeSequence was performed *via* the included utility. ARG reconstruction was about 20 times slower than for Moonshine across mutation rates. Additionally, Relate failed to map 2 and 1 mutations for $\mu =5\times 10^{-9}$ and $\mu =1\times 10^{-8}$ , respectively.

SINGER outputs multiple ARGs using a MCMC scheme and requires choosing a thinning interval as well as a burn-in period. For the former, we used the default of discarding 20 ancestries between samples. Under this regime, we sampled 2000 ARGs and assessed convergence, which was attained for every parameter of interest after about 1,000 iterations. The last 200 ARGs were stored for analysis. We used the included Python script for conversion to TreeSequence. We had to manually reorder mutations due to a known bug of the script when dealing with flipped SNPS. Although we used the recommended prior probability of correct polarization of 0.99, some flips did occur, about 0.8 by ARG on average for $\mu =2\times 10^{-8}$ . Reconstruction of a single ARG was between 58 and 21 times slower than for Moonshine with the difference decreasing as the mutation rate (and hence the number of markers) increases. Note that this does not account for ancestries discarded during the burn-in period.

**FIGURE 15:**
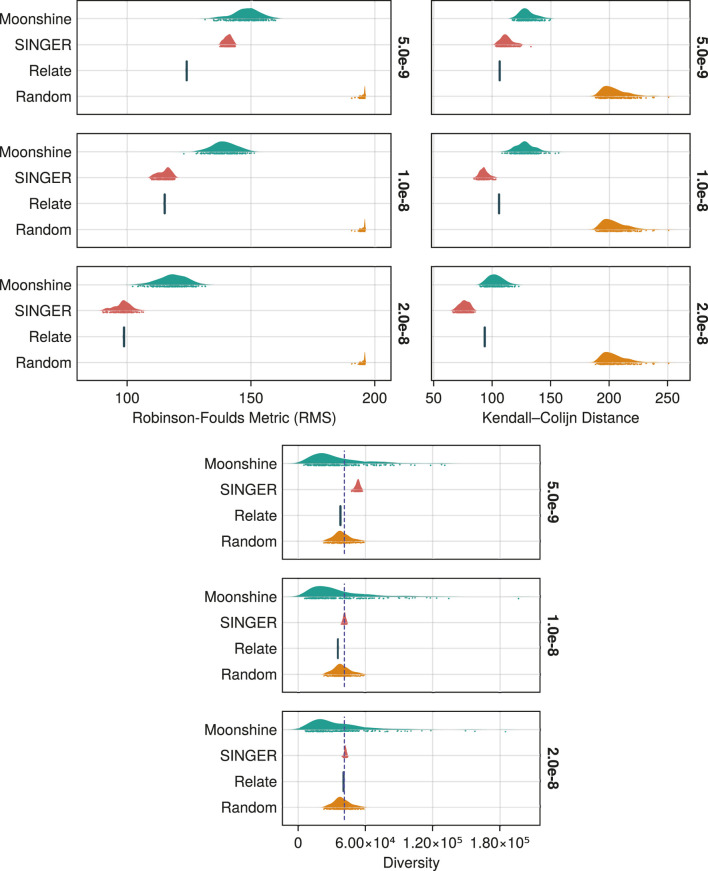
Accuracy of ARG reconstruction. Mutation rate is indicated on the right of the figures. An estimate of the density is provided for each metric for both Moonshine and SINGER. Since Relate is deterministic, the unique computed value is indicated by a vertical line. The dashed line in the bottom plot marks the true diversity of the simulated ARG.

We compared ARGs using three metrics: the Robinson-Foulds (RF) distance Robinson and Foulds (1981), the Kendall–Colijn (KC) distance Kendall and Colijn (2016) and the branch-length-based diversity (i.e., average pairwise coalescence times). The first two metrics measure accuracy in topology reconstruction while the last one evaluates inference of pairwise coalescence times. Since the RF metric is only defined for trees, we evaluated it on a regular grid of 25 positions and reported the root mean squared error (RMSE) with respect to the original ARG. Each of these quantities was measured using the corresponding methods in tskit. To establish a baseline, we compared 200 simulated ARGs with the original for each metric. We employed the same parameters as we did for the original ARG. Results are presented in Figure 15. The baseline is the “Random” category.

Moonshine behaves as expected with respect to topological metrics. In both cases, reconstruction accuracy increases with the number of markers. The same is true for SINGER and Relate, which are slightly more accurate than Moonshine. The diversity of ARGs reconstructed by Moonshine and Relate is largely unaffected by the number of markers. In the same regard, performance of SINGER is generally very good and improves as the number of markers increases.

## 3 Results

### 3.1 Results

All simulations were performed with Moonshine version 0.3.9 and Julia 1.11.3 on AMD EPYC 9654 processors. Execution time was measured using the @timed macro. To reduce variability due to the execution environment and differences between ARGs, each reported measurement is the minimum of three runs, averaged over five graphs. Haplotypes are generated by msprime using StandardCoalescent and BinaryMutationModel as the ancestry and mutation models, respectively. Per-locus mutation and recombination rates were both set to $10^{-8}$ . An effective population size of $10^{4}$ was specified. The calls to msprime.sim_ancestry and msprime.sim_mutations were made through the Moonshine interface to msprime. Moonshine 0.3.3 does not implement the recoalescence latitude generation procedures described in Subsection 2.2.5. In particular, its algorithm for wild events is less performant. Consequently, we expect the performance of versions 0.3.6 onwards, which implement the time-scale transformation approach, to be slightly better when using the default grid.

All the code used for the simulation studies, as well as the raw data, is publicly available on Codeberg (https://codeberg.org/ptrk/moonshine.jl-papers). For convenient reproducibility, code to execute each simulation presented and produce related figures is grouped in a single Pluto notebook. As the simulations are computationally intensive and require considerable time to complete, it may be more convenient to run them on more powerful machines such as those found in a computer cluster. For that purpose, the notebook can be executed as a standalone Julia script. Figure generation steps will be skipped when doing so; they can be run locally later on.

Raw simulation results are presented below. Numbers of markers and recombination events are averaged over constructed ARGs.

## 4 Discussion

We have described a fast and efficient algorithm for ancestral recombination graph inference and its Julia implementation *via* the Moonshine package. In addition to the exact procedure, an approximate scheme based on user-defined genomic windows is available, which reduces inference time. Inference is grounded in a restricted version of the coalescent-with-recombination distribution rather than heuristics. We have also presented a flexible algorithm for coalescent tree construction that can accommodate a wide range of metrics on haplotypes, exactly or approximately, while avoiding numerical instabilities.

One of the main drawbacks of Moonshine is the need for high-quality phased and polarized data. This limits the applicability of our method, as such data is relatively scarce in practice. Moreover, our approach is limited to binary markers and assumes non-recurrent mutations. Some of these limitations are more significant than others. For instance, the data structure used for storing haplotypes is fairly straightforward to adapt to multiallelic markers, and minor modifications to our algorithms would be sufficient to account for simple, non-recurrent mutation models, which mainly involve the existence of an absorbing state.

As of version 0.3.3, these algorithms are for the most part mature. No significant leap in inference time or memory usage is to be expected in the near future. Our goal for version 1.0.0 is to improve real-world usability. Specifically, the API reference needs to be completed to make implementing alternative models a more pleasant experience. General documentation and guides also need to be improved for the general user. Both are publicly available at https://patrickfournier.ca/software/documentation/moonshine?

As of today, version 0.3.4 and later are compatible with files encoded in the variant call format (VCF) through the VCFTools.jl package (Chu et al., 2023). We plan to support FASTA/FASTQ and sequence/binary alignment map format (SAM/BAM) *via* the FASTX.jl and XAM.jl packages, respectively. Along with compatibility with these input formats, we plan to support missing markers by treating them as non-ancestral material, which is the approach taken by Lyngsø et al. (2005); Minichiello and Durbin (2006); Ignatieva et al. (2021).

Moonshine is available on Julia’s general registry and can be easily and quickly obtained using standard facilities. In addition, we plan to package it in two application containers. One will include the Pluto notebook (van der Plas, 2025) package and graphical utilities such as Makie.jl (Danisch and Krumbiegel, 2021). Its goal is to reduce the burden associated with ARG inference for practitioners. The other container will be more minimal, including only the minimum required to deploy Moonshine *via* an orchestration system such as kubernetes. Our hope is to facilitate the creation of high-performance computation clusters in small-to-medium environments.

## Data Availability

The original contributions presented in the study are included in the article/Supplementary Material, further inquiries can be directed to the corresponding author.

## Appendix

**Secretary sampler**

The following result gives the probability that a run of the secretary sampler is exact.

Lemma 1 Let K be the index of the sample returned by a run of the secretary sampler on a vector of size m with threshold $t_{0}$ , $t_{m}=\left\lfloor mt_{0}\right\rfloor$ , $p_{i}=p_{ab_{i}}$ , and $K_{t_{m}}={\text{arg max}}_{i=1}^{t_{m}}\left\{g_{i}+\log p_{i}\right\}$ . Let $\hat{K}$ be the index of the correct sample. Then,

$$\mathrm{Pr}\left(K=\hat{K}\right)=\frac{1}{\overset{m}{\underset{i=1}{\sum }}p_{i}}\left(\overset{t_{m}}{\underset{i=1}{\sum }}p_{i}+p_{K_{t_{m}}}^{\hat{K}-t_{m}-1}\left(\overset{m}{\underset{i=t_{m}+1}{\sum }}p_{i}\right)\overset{\hat{K}-1}{\underset{i=t_{m}+1}{\prod }}{\left(p_{K_{t_{m}}}+p_{i}\right)}^{-1}\right).$$

Proof. By the formula of total probability,

$$\mathrm{Pr}\left(K=\hat{K}\right)=\mathrm{Pr}\left(K=\hat{K}\;|\;\hat{K}\leq t_{m}\right)\mathrm{Pr}\left(\hat{K}\leq t_{m}\right)+\mathrm{Pr}\left(K=\hat{K}\;|\;\hat{K}>t_{m}\right)\mathrm{Pr}\left(\hat{K}>t_{m}\right).$$

Since the algorithm visits every element with an index below $t_{m}$ , $\mathrm{Pr}\left(K=\hat{K}\;|\;\hat{K}\leq t_{m}\right)=1$ . Given that $\hat{K}$ is distributed as a categorical random variable, we obtain

$$\mathrm{Pr}\left(\hat{K}\leq t_{m}\right)=\frac{\sum_{i=1}^{t_{m}}p_{i}}{\sum_{i=1}^{m}p_{i}}$$

$$\mathrm{Pr}\left(\hat{K}>t_{m}\right)=\frac{\sum_{i=t_{m}+1}^{m}p_{i}}{\sum_{i=1}^{m}p_{i}}.$$

It only remains to show

$$\mathrm{Pr}\left(K=\hat{K}\;|\;\hat{K}>t_{m}\right)=p_{K_{t_{m}}}^{\hat{K}-t_{m}-1}\overset{\hat{K}-1}{\underset{i=t_{m}+1}{\prod }}{\left(p_{K_{t_{m}}}+p_{i}\right)}^{-1}.$$

By pairwise independence of the sequence $g_{1},\ldots ,g_{m}$ , we obtain, after grouping similar terms, the following expression:

$$\mathrm{Pr}\left(K=\hat{K}\;|\;\hat{K}>t_{m}\right)=\overset{\hat{K}-1}{\underset{i=t_{m}+1}{\prod }}\mathrm{Pr}\left(g_{i}-g_{K_{t_{m}}}\leq \log p_{K_{t_{m}}}-\log p_{i}\right)$$

Since $g_{i}$ and $g_{K_{t_{m}}}$ follow a standard Gumbel distribution, their difference is distributed as a standard Logistic distribution. In consequence, the probability of interest is nothing more than the CDF of this distribution. Routine simplifications complete the proof.
